# Editorial

**Published:** 1872-05

**Authors:** 


### Editorial.

***Illinois State Medical Society.***

At the time of going to press we have advices from this Society, assembled at Rock Island, to the effect that the meeting is well attended, and several valuable papers have been presented. The following officers have been elected for the ensuing year:

*President*—D. W. Young, M.D., Aurora.
1*st Vice-President*—Thos. D. Washburn, M.D., Hillsboro.
2*nd Vice-President*—T. C. Hotz, M.D., Chicago.
*Permanent Secretary*—T. D. Fitch, M.D., Chicago.
*Assistant Secretary*—R. D. Bradley, M.D., Bloomington.

Bloomington was selected as the place for the next annual meeting.

***The American Journal of Obstetrics***

And Diseases of Women and Children—edited by B. F. Dawson, M.D., and published in New York by Wm. Baldwin & Co.; a quarterly, at $5 a year, (specimen copies, 50 cents)—is a periodical that we take especial pleasure in commending to our readers. It is elegantly gotten up; in its editorial conduct high-toned, and full, from cover to cover, of valuable and interesting original and carefully selected matter. It is so rare to find an organ of this specialty that we can heartily approve, that it is a novel sensation to be able to write as we now do. Please make a note of it accordingly.

